# Personalized medicine using DNA biomarkers: a review

**DOI:** 10.1007/s00439-012-1188-9

**Published:** 2012-07-01

**Authors:** Andreas Ziegler, Armin Koch, Katja Krockenberger, Anika Großhennig

**Affiliations:** 1 Institut für Medizinische Biometrie und Statistik, Universität zu Lübeck, Universitätsklinikum Schleswig–Holstein, Campus Lübeck, Maria-Goeppert-Str. 1, 23562 Lübeck, Germany; 2 Zentrum für Klinische Studien, Universität zu Lübeck, Lübeck, Germany; 3 Institut für Biometrie, Medizinische Hochschule Hannover, OE 8410, 30625 Hannover, Germany

## Abstract

Biomarkers are of increasing importance for personalized medicine, with applications including diagnosis, prognosis, and selection of targeted therapies. Their use is extremely diverse, ranging from pharmacodynamics to treatment monitoring. Following a concise review of terminology, we provide examples and current applications of three broad categories of biomarkers—DNA biomarkers, DNA tumor biomarkers, and other general biomarkers. We outline clinical trial phases for identifying and validating diagnostic and prognostic biomarkers. Predictive biomarkers, more generally termed companion diagnostic tests predict treatment response in terms of efficacy and/or safety. We consider suitability of clinical trial designs for predictive biomarkers, including a detailed discussion of validation study designs, with emphasis on interpretation of study results. We specifically discuss the interpretability of treatment effects if a large set of DNA biomarker profiles is available and the number of therapies is identical to the number of different profiles.

## Introduction

Genetic variation contributes to both disease susceptibility and treatment response. Genome-wide association studies (GWAs) have enabled rapid discovery of genetic variants contributing to the pathogenesis of complex genetic diseases (Manolio 2010), as well as detection of many pharmacogenetic markers (Link et al. 2008; Verschuren et al. 2012). The driving hope of these major advances in genetic epidemiology is that promotion of personalized medicine will improve medical decision-making.

Although use of the term personalized medicine is often limited to the identification of the optimal drug and the optimal dosage for a subgroup of patients, current personalized medicine applications are far more broad, and might include situations of withholding treatment, preventive interventions, or targeted treatment options for individual patients. In prostate cancer, for example, DNA biomarker tests may be used to determine whether treatment may be safely delayed for a period of watchful waiting. If the tumor is demonstrated due to lack of genes causing an aggressive form of the cancer, it may remain stable for decades, and the need for radical surgical resection with subsequent radio- or chemotherapy may be obviated (Kroll 2008). In contrast, in other instances, genetic profiles may be used to determine preventive interventions. This approach is already used for some forms of hereditary cancer, in which individual genetic testing is the basis for deciding upon specific, sometimes very radical interventions such as preventive surgery (Kroll 2008).

Beyond treatment schemes that are applied identically across large subgroups of patients—to which some authors have applied the distinct term stratified medicine (Trusheim et al. 2007)—other personalized medicine applications offer targeted treatment options for individual patients. Anti-inflammatory therapies, for example, such as anti TNF, anti IL-6, or anti IL-1β, are thought to be effective in inflammatory diseases, such as Crohn’s disease (Buchner et al. 2011; Cottone et al. 2010). Other molecules seem suitable for use as anti-inflammatory therapy as well. To choose one or a combination of different anti-inflammatory therapies, the physician might first obtain the genetic profile of a patient by sequencing. Depending on the individual DNA profile, the physician might select the anti-inflammatory combination therapy, of which there are many.

Though foundational to personalized medicine, biological markers, biomarkers for short, are diversely defined in the literature. Gallo et al. (2011) summarize some of the definitions, and observe that the most commonly adopted definition states that “a biomarker is any substance or biological structure that can be measured in the human body and may influence, explain or predict the incidence or outcome of disease”. However, it is a matter of debate whether the qualification that biomarkers must be measured in the human body is a reasonable limitation. A related definition has been given by Gallo et al. (2011) who define “biomarkers as any substance, structure or process that can be measured in biospecimen and which may be associated with health-related outcomes”. From our perspective, this definition is too general and should include a specific association with a health or clinical outcome. Our preference, therefore, is for the definition developed by the Biomarkers Definitions Working Group (2001): “A biomarker is a characteristic that is objectively measured and evaluated as an indicator of normal biological processes, pathogenic processes or pharmacologic responses to a therapeutic intervention.” Although more than a decade old, this definition is comprehensive and sufficiently broad to capture the full range of current biomarker applications, described in detail in the Table 1.

**Table 1:** Important terms in biomarker studies

Term	Explanation
Biomarker	A characteristic that is objectively measured and evaluated as an indicator of normal biological processes, pathogenic processes, or pharmacologic responses to a therapeutic intervention (Biomarkers Definitions Working Group 2001).
Cancer biomarker	A biomarker that is present in tumor tissue or serum and includes many different molecules, such as DNA, mRNA, or proteins. Tumor biomarkers are measured in tumor tissue, and tumor DNA biomarkers are measured from tumor tissue.
Clinical endpoint	A characteristic or variable that reflects how a patient feels, functions, or survives (Biomarkers Definitions Working Group 2001).
Companion endpoint	A biomarker that is essential to the efficacy and safety of a corresponding therapeutic product (Food and Drug Administration 2011).
Copy number variant (CNV) biomarker	A biomarker of genomic variation in which blocks of DNA are missing or for which multiple copies exist.
Diagnostic biomarker	A biomarker that relates to the diagnosis or severity of disease. The most important diagnostic biomarkers are screening biomarkers.
Disease biomarker	A biomarker that relates to a clinical endpoint or measure of disease (Kroll 2008).
DNA biomarker	A germline biomarker, such as SNPs, STRs, deletions, insertions, or other variation on the DNA sequence level.
Efficacy biomarker	A biomarker that predicts a beneficial effect of a given treatment (Kroll 2008).
Epigenetic biomarker	A biomarker that measures epigenetic alterations, such as DNA methylation, histone methylation, histone acetylation, microRNAs, or other non-coding RNA (Bock 2009).
Monitoring biomarker	A biomarker to monitor efficacy or side effects of a drug treatment.
Prognostic biomarker	A biomarker that predicts the likely course of disease in a defined clinical population under standard treatment conditions.
Prediction model	A predictive test including multiple markers.
Predictive biomarker	A biomarker that forecasts the likely response to treatment (Buyse et al. 2011). Treatment response may be measured either as efficacy or as safety.
Predictive test	Two definitions exist in the literature a test of test of probability for an individual to develop a disease; alternatively, a test which discriminates between individuals who will develop a disease and those who will not (Janssens and van Duijn 2008).
Risk prediction	The generation or validation of models which make a prognosis for developing a disease or the prognosis for attaining a clinical endpoint.
Safety biomarker	A biomarker that indicates adverse response to a treatment (Sistare et al. 2010). Toxicity biomarkers are special cases of safety biomarkers.
Screening biomarker	A biomarker to discriminate between healthy individuals and those in an early stage of the disease (Kroll 2008), ideally while subjects are asymptomatic.
Staging biomarker	A biomarker that distinguishes between different stages of chronic disease (Kroll 2008).
Stratification biomarker	See predictive biomarker.
Surrogate biomarker	A biomarker that is regarded as a valid substitute for a clinical endpoint. A surrogate endpoint is expected to predict clinical benefit (or harm or lack of benefit or harm) (Biomarkers Definitions Working Group 2001; Kroll 2008).
Target biomarker	A biomarker that reports interaction of the drug with its target (Kroll 2008).
Toxicity biomarker	A biomarker that reports to the toxic effect of a drug on an in vitro or in vivo system (Kroll 2008).

In personalized medicine, it is necessary to distinguish between prognostic and predictive biomarkers. Following Buyse et al. (2011), the difference is that prognostic biomarkers help in predicting the progress of the disease, while predictive biomarkers are connected with the response to a treatment.

Having clarified fundamental terms, we will proceed to a discussion of similarities and differences of biomarkers, illustrate current uses of some of these biomarkers, and outline specific aspects of diagnostic, prognostic, and predictive biomarker studies. Finally, we will discuss various study designs for the validation of predictive biomarkers in detail.

## DNA biomarkers, DNA tumor biomarkers, and general biomarkers

Genetic information, coded within DNA, requires stability because DNA directs the production of proteins required for the cell structure and function of cells over a lifetime. Some authors state that DNA is stable over an individual’s lifetime (Hicks and Coquoz 2009), and biomarkers explicitly representing this stability are termed “DNA biomarkers” in the discussion which follows. Single nucleotide polymorphisms (SNPs), short tandem repeats (STRs), deletions, insertions, or other variation on the DNA sequence level are among this group. Due to the availability of high-throughput molecular biological facilities, SNPs are the most commonly used type of DNA variation. In most applications SNPs are diallelic, resulting in three possible genotypes.

Cancer is a disease that involves changes to the DNA at the cellular level, and these changes can be measured in the tumor. Distinct from DNA biomarkers outlined above, we will use the term “DNA tumor biomarkers” to indicate biomarkers specific to cancerous tumors. Typically, only the presence or absence of a mutation in a gene is determined.

Finally, we use the term “general biomarkers” for all other forms of biomarkers, such as RNA, protein, or metabolite measurements which can be measured in biofluid, tissue, or even cell lines. While most general biomarkers share the property of being quantitative with positive measurement values, both DNA biomarkers and DNA tumor markers are discrete in nature (Table 2). Nevertheless, when used in the diagnostic process, thresholds need to be introduced for all types of biomarkers to relate biomarker measurements to clinical decision-making.

**Table 2:** Differences between DNA biomarkers, DNA tumor biomarkers, and general biomarkers

Characteristic	DNA biomarker	DNA tumor biomarker	General biomarker
Level of measurement	Discrete. In SNPs, one of three different genotypes is observed per subject, in general	Discrete. In general, the measurement is whether a specific gene is mutated or not	Continuous. RNA, protein, and metabolite concentrations may take almost any continuous positive value
Stability, reproducibility	Yes	Not necessarily as different mutations may be present in different parts of the tumor	Only at one specific time point
Suitable for therapy monitoring	No	Yes	Yes
Suitable for pharmacodynamics	No	Yes	Yes
Suitable as surrogate marker	No	No, in general	Yes
Complexity of measurement	Low	High	High
Time required for measurement (includes drawing and preparation of sample)	Low	High	High
Time of measurement	Does not have to be specified	Needs to be specified in advance	Needs to be specified in advance
Retrospective validation of biomarker	Yes	No	No
“Durability” of the final biomarker test	Short- to long-term; multimarker sets may be already obsolete at start of prospective study	Mid-term to long-term	Mid-term to long-term
Study design	Retrospective or prospective	Prospective only	Prospective only

DNA biomarkers are generally measured in the blood, tumor DNA biomarkers are measured in tumor tissue, general biomarkers may be measured in biofluid, tissue, or cell lines

Important differences between DNA biomarkers and DNA tumor or general biomarkers stem from the fact that DNA is stable over the entire lifetime (Table 2). DNA biomarkers are reproducible, can be measured at any point in time, and may be used in both prospective and retrospective studies. Specifically, DNA biomarkers can be prospectively validated in biobanks, i.e., in studies, where clinical information has already been collected. The authors stress that they would not call such kind of study a prospective one.

In general, DNA biomarkers are simpler to measure than DNA tumor or general biomarkers. Sample drawing, handling and storage protocols are generally also simpler for DNA biomarkers, and laboratory time and cost for measurement is lower. Nevertheless, DNA biomarkers are not without disadvantages.

First, as they do not vary over lifetime, they cannot be used for therapy monitoring, pharmacodynamics, or as surrogate markers. A second general problem is “durability”, as the rate of discovery of new DNA biomarkers is frequently more rapid than their product cycles. By the time, a DNA multimarker test has successfully passed all steps for marketing approval, including refunding by health insurance companies, it may already be rendered scientifically obsolete by newly discovered DNA biomarkers with seemingly better performance.

Pertinent differences between DNA tumor biomarkers and general biomarkers (Table 2) include the fact that DNA tumor biomarkers generally cannot serve as surrogate markers—while general biomarkers often can—and the fact that DNA tumor biomarkers can show greater variation, depending on how they are measured. If a DNA tumor biomarker is measured through biopsy, for example, it is possible for one biopsy probe to be tumor free, while another contains tumor cells, and this difference may result in different DNA tumor biomarker results. In contrast, if a general biomarker is measured, for example, from plasma, it is generally stable for the time point of measurement.

## Examples for current use of biomarkers

In this section, we illustrate current applications of biomarkers for diagnosis, prognosis, and prediction in personalized medicine. Details of the examples outlined in this section are provided in Table 3, together with additional examples of general biomarkers, and in particular, DNA biomarkers and DNA tumor biomarkers, in current use.

**Table 3:** Examples for biomarkers in current use

Name	Type	Range of application	Commercial use	Indication	Time of measure	Outcome	Reference
BluePrint^®^	DNA tumor	Predictive	Yes	Breast cancer	Known diagnosis, after surgery	Reaction of individual therapies	Krijgsman et al. (2012)
*EGFR*	DNA tumor	Predictive	PCO	Advanced non-small-cell lung cancer	Known diagnosis, before first-line therapy	EGFR TKI or chemotherapy	Keedy et al. (2011)
*IL28B*	DNA	Predictive	–	Hepatitis C virus 1 (HCV-1)	Known diagnosis, before treatment	Response to treatment with pegylated interferon (Peg-IFN) combined with ribavirin (RBV)	Holmes et al. (2011)
*K-RAS*	DNA tumor	Prognostic	PCO	Advanced colorectal cancer	Known diagnosis, before chemotherapy	Treatment with cetuximab yes or no	Karapetis et al. (2008)
MammaPrint^®^	DNA tumor	Prognostic	Yes	Breast cancer	Known diagnosis, after operation	Precise stage of tumor, aggressivity of tumor	Buyse et al. (2011)
OncoTypDX^®^	DNA tumor	Predictive/prognostic	Yes	ER-positive, HER2-negative breast cancer, colon cancer	Known diagnosis, after operation	Chemotherapy recommended yes/no	Buyse et al. (2011)
Point-of-care tests: RheumaChec CCPoint assay	General	Diagnostic (screening)	Yes	Rheumatoid arthritis (RA)	Before first symptomatic	Earlier therapy for RA	Egerer et al. (2009)
*SLCO1B1*	DNA	Predictive	–	Myocardial infarction	Known diagnosis, before treatment	Reduction of statin doses cause of statin-induced myopathy, security monitoring	Link et al. (2008)

*EGFR* epidermal growth factor receptor, *EGFR TKI* EGFR tyrosine kinase inhibitor, *ER* estrogen receptor, *HER2* hormone receptor,* K-RAS* Kirsten rat sarcoma viral oncogene homolog,* PCO* provisional clinical opinion of the American Society of Clinical Oncology

Diagnostic biomarkers are biomarkers used to determine the diagnosis or severity of a disease. The most important within this group are screening biomarkers (Table 1), which are used to discriminate between healthy individuals and those in an early stage of a disease. For example, the commercially available point-of-care tests (POCT) Rheuma-Chec and CCPoint, test serum for antibodies to mutated citrullinated vitmentin (MCV) or citrullinated peptides/proteins (anti-CCP antibodies), in order to screen for rheumatoid arthritis in non-symptomatic, healthy persons (Egerer et al. 2009).

If a diagnosis is known, prognostic biomarkers help to predict the likely course of disease in a defined clinical population under standard treatment conditions. For example, MammaPrint^®^, a DNA tumor biomarker for breast cancer prognosis is used following surgery to indicate whether risk of metastasis is low or high, and guide physicians to determine the best kind of treatment for the individual patient. Such therapy guidance requires the validation of the predictive capability of the biomarker. In fact, in the United States, MammaPrint^®^ is cleared by the Food and Drug Administration (FDA) as an in vitro diagnostic multivariate index assay (IVDMIA) (Slodkowska and Ross 2009). In Germany, as of 2012, some health insurance companies will pay the cost of the test for particular cases.

Predictive biomarkers predict the likely response of a patient to a special treatment in terms of efficacy and/or safety and thus support clinical decision-making (Table 1). For example, GWAs conducted by three independent groups from North America, Australia/Northern Europe, and Japan (Holmes et al. 2011) demonstrated that the *IL28B* gene is a strong indicator for response to standard treatment in patients with hepatitis C virus-1 (HCV-1) infection. Further, there is evidence for population stratification in the *IL28B* gene, such that treatment response varied across different ethnic groups. Specifically, Caucasians with the “good response” genotype were more likely to benefit from treatment. This fact is also mentioned in a Provisional Guidance for the treatment of HCV (Thomas et al. 2012), which notes that treatment with special inhibitors is expected to be less efficacious in persons with the “non-response” genotype, or African ancestry.

While some biomarkers have already been approved by the FDA, the use of others has been recommended in clinical guidelines such as the Provisional Clinical Opinion from the American Society of Clinical Oncology (ASCO), which publishes clinical direction based on potentially practice-changing data from major studies. A recent example of this is a test for epidermal growth factor receptor (*EGFR*) mutation in patients with advanced non-small-cell lung cancer, which determines whether or not the first-line EGR tyrosine kinase inhibitor therapy is indicated (Keedy et al. 2011).

## Phases of diagnostic biomarker studies

The four different phases in the drug development process are generally always fixed. Regulatory approval is required before a new drug can enter the market. In the preclinical program, safety, pharmacology and pharmacodynamics are investigated in animal models. The first three clinical phases establish pharmacokinetics, pharmacodynamics, the required dose, clinical efficacy and benefit/risk. Following marketing approval, effects on morbidity and mortality endpoints, or relative effectiveness are investigated in the fourth phase (European Medicines Agency 1998b).

Development programs of diagnostic tests undergo a similar process (European Medicines Agency 2009). In phase I, it must be demonstrated that the diagnostic test may be safely applied to humans, and that the technical properties are well understood, such as mode of application, reproducibility, etc. In phase II, the test is applied to individuals known to be either diseased or “healthy”, in order to obtain initial estimates of sensitivity and specificity. Phase III studies are the so-called validation studies, in which the diagnostic test and subsequent diagnostic workup is carried out in the same manner and setting as it would be in later clinical and diagnostic practice. Finally, phase IV diagnostic studies are performed to investigate whether the application of the test leads to a measurable improvement of the clinical outcome in a broader population.

This phase concept is oriented towards marketing approval of diagnostic agents that are intended for in vivo administration, such as radiopharmaceuticals or contrast agents for use in imaging techniques, such as magnetic resonance imaging. In our own projects, we have found an extended phase approach, summarized in Table 4, to be more helpful from a developmental perspective for diagnostic or prognostic biomarkers (compare the phase models of Pepe 2003, p. 215, Pepe et al. 2001, Baker et al. 2006, and Riley et al. 2009).

**Table 4:** Phases of diagnostic or prognostic biomarker studies

Phase	Diagnostic and prognostic biomarkers		Typical sample sizes
	Description	Aim of study	
Ia	Discovery	Identification of promising biomarkers	10–100
Ib	Assay development, assay validation	Define and optimize analytical process into robust, reproducible, and valid device	10–100
Ic	Retrospective validation	Clinical assay detects disease; development of first algorithm for combination test	50–500
II	Retrospective refinement	Validation of early detection properties of biomarker (set); development and/or refinement of algorithm(s) for combination tests	100–1,000
III	Prospective investigation	Determination of diagnostic accuracy (sensitivity, specificity) in the situation of clinical routine	200–1,000
IVa	Randomized controlled trial	Quantification of effect of making the biomarker information available to the doctor to reduce disease burden	200–1,000
IVb	Health economics study	Quantification of cost-effectiveness	Strongly depends on clinical consideration of clinical risk

In phase I of this extended approach, we delineate three distinct sub-phases: discovery (Ia), assay development and validation (Ib), and retrospective validation (Ic). The discovery study (phase Ia) is typically performed using high-throughput technologies. For DNA biomarkers, the discovery phase might be a GWAs, a genome-wide meta-analysis or even a whole genome sequencing study. In proteomics, this discovery phase might consist of expression clone arrays containing tens of thousands of recombinant human proteins (for a recent application to prostate cancer, see Massoner et al. 2012) or multiplex protein antigen analysis on a Luminex platform (Linkov et al. 2009).

The high-throughput arrays or whole genome sequences will not be the final product for the diagnostic test to be released on the market. Therefore, in phase Ib the identified biomarkers are adapted to a laboratory setting which might be integrated into clinical routine. The complementary technology introduced in this phase is generally more reliable and precise, i.e., its coefficient of variation is lower and typically has reduced bias. For DNA biomarkers, this phase combines assay transfer with a better-suited laboratory platform and the choice of the DNA marker(s) to be typed on this platform. In proteomics, this might represent the development of an ELISA, such as the ELISA for AGR2 in voided urine for detecting secreted prostate cancer (Wayner et al. 2011).

Following the discovery phase and the change in the specific laboratory technology, a first retrospective validation is generally performed (phase Ic) to determine whether the results from the imprecise high-throughput technology still hold true. It is important to note that the sample size in this first retrospective validation is higher than in the initial high-throughput search (Table 4, last column).

Subsequent to retrospective validation, multimarker models are developed, either as part of phase Ic, with the initial cohort of patients, or as part of phase II, in a retrospective study using patients different from those of phase I. Whether initially developed in phase I or II, this model will always undergo refinement with the second group of patients. Nevertheless, a prospective investigation in phase III may also often be necessary to obtain reliable estimators for sensitivity and specificity of DNA biomarker tests, or, for prognostic biomarkers, to attain reliable estimates of the prognosis, e.g., through the use of Brier scores.

To assess the impact of the diagnostic DNA biomarker on patient management, a randomized controlled trial is conducted (in phase IVa), following marketing approval demonstrating that treating doctors’ knowledge of the test result improves patient outcome. This phase mimics the development of a predictive biomarker (see next section). Finally, health economics studies may be carried out in order to assess cost-effectiveness of the diagnostic test (phase IVb).

A challenging element of this extended phase model for diagnostic and prognostic biomarkers is the term “prospective validation” for phase III. Because germline DNA is assumed to be stable, the prospective investigation of an assay in an already ascertained biobank may be considered to be prospective. However, it must be definitively demonstrated that the biobank has not been used before, because multiple testing will arise if a biobank is used for biomarker validation more than once. Proper definition of standards as to when biobanks may be used for prospective validation of DNA biomarkers is complex. The situation becomes even more complex when considering DNA tumor biomarkers or general biomarkers, as these biomarkers may change over time such that the time point of sample drawing may also be crucial. Furthermore, different sample handling and storage protocols as well as age of samples may have an effect not only on biomarker quality but also on biomarker levels (Table 2).

Irrespective of the phase model used (for other alternatives, see, e.g., Zhou et al. 2001, p. 61), assay sensitivity—the technical and analytical validity of the biomarkers—is a mandatory requirement. Given high assay sensitivity, the problem of missing values can be ignored because they are assumed to occur only due to random technical failure and not any systematic process.

## Validation of predictive biomarkers used as companion tests

As outlined in Sect. “Examples for current use of biomarkers”, above, and detailed in Table 3, a number of biomarkers have recently been identified to predict treatment outcome, and some have proven particularly effective in resolving unconvincing or ambiguous clinical trial results. For example, in the multicenter IPASS (Iressa Pan-Asia Study) trial of advanced non-small cell lung cancer (NSCLC), patients treated with either gefitinib or carboplatin plus paclitaxel, progression-free survival curves of both treatment groups crossed in the general population of patients (1,200 previously untreated East-Asian non-smokers or former light smokers). When treatment groups were stratified for tumors bearing epidermal growth factor receptor gene (*EFGR*) mutation, however, important differences emerged: *EGFR*-positive patients’ progression-free survival was consistently longer in the gefinitab treatment group, while *EGFR*-negative patients’ progression-free survival was longer in the carboplatin–paclitaxel group (Mok et al. 2009). As noted in Sect. “Examples for current use of biomarkers”, the strength of the relationship was sufficiently compelling that the American Society for Clinical Oncology has since recommended that patients with NSCLC should have their tumors tested for *EGFR* mutations to determine the most appropriate first-line therapy (Keedy et al. 2011).

In most instances these biomarkers have been identified after the conduct of phase III trials that were intended to provide the pivotal evidence about efficacy and positive benefit/risk of an experimental treatment required to justify market approval. An important consequence of the retrospective nature of these investigations was that the appropriateness of the biomarker validation had to be assessed on a case-by-case basis.

In July 2011, the FDA issued a draft guidance for industry for “in vitro companion diagnostic devices”, which are predictive biomarkers essential for safe and effective use of a corresponding therapeutic product (Food and Drug Administration 2011). The draft guidance anticipated three uses of companion diagnostics:

1. Identification of patients most likely to benefit from a particular therapeutic product;
2. Identification of patients likely to be at increased risk for adverse reactions to a therapeutic product, and;
3. Monitoring treatment response to adjust treatment in order to improve efficacy and safety.

If either treatment effect or tolerability differs with respect to the companion diagnostic, the diagnostic test can be used to refine the patient population. DNA biomarkers and DNA tumor biomarkers cannot be used for treatment monitoring for obvious reasons. However, they can be used for aspects 1 and 2.

The FDA guideline suggests that because companion diagnostics provide critical information for the appropriate use of drugs, they require validation as part of the evaluation of efficacy of the experimental treatments, and information about the diagnostic is reflected in the drugs’ labeling. The use of biobanks for prospective validation, therefore, might play a role for already approved drugs, if biomaterial allows systematic investigations into improved efficacy or safety in biomarker-defined subgroups. Within the pharmaceutical industry, such research will become increasingly important to pharmacovigilance and post-marketing surveillance of drug use. Valid conclusions are, however, not possible if material for genotyping is available for only so-called convenience samples (Wang et al. 2010).

At the end of the development program, clinical data should substantiate that efficacy and/or benefit/risk of the experimental treatment as compared to control is substantially larger in patients who test positive with the companion diagnostic test than in those who test negative. If the experimental treatment shows comparable positive effects in test-negative patients and in test-positive patients, the diagnostic test would not be required, and the new treatment might simply be provided to all patients. Thus, diagnostic tests are only of value if clinical data or ethical reasons support that treatment be withheld in test-negative patients.

In the context of clarifying specific circumstances in which a new drug might be used appropriately, information from test-negative patients must be available in order to justify population refinement, and the costs that use of the biomarker prior to treatment will incur on the health insurance system. It may be a matter of debate, or even discriminative power of the companion biomarker, whether this information might be obtained from a phase II clinical trial, or whether it should be obtained from the same trial used to demonstrate efficacy of the experimental treatment. Obviously, there is no need to demonstrate in statistical terms that the experimental treatment does not have advantages over control in test-negative patients. However, if in the same trial there is no clear trend towards superior efficacy or improved benefit/risk, especially in a phase II trial of limited size, the usefulness of the companion biomarker might be questioned.

Official regulatory guidance about appropriate study designs to validate companion diagnostics together with new drugs is still sparse, although both the FDA and European Medicines Agency (EMA) have established procedures, in which applicants may negotiate the amount of evidence required for marketing authorization in this setting (European Medicines Agency 2012).

## Study designs for predictive biomarkers

Pioneering work on clinical trial designs for predictive biomarker validation has been performed by Sargent et al. (2005), Mandrekar and Sargent (2009a, b). More recently, Buyse et al. (2011), in their seminal review, discuss 10 different study designs for co-development of an experimental drug and a companion diagnostic test.

**Fig. 1:**
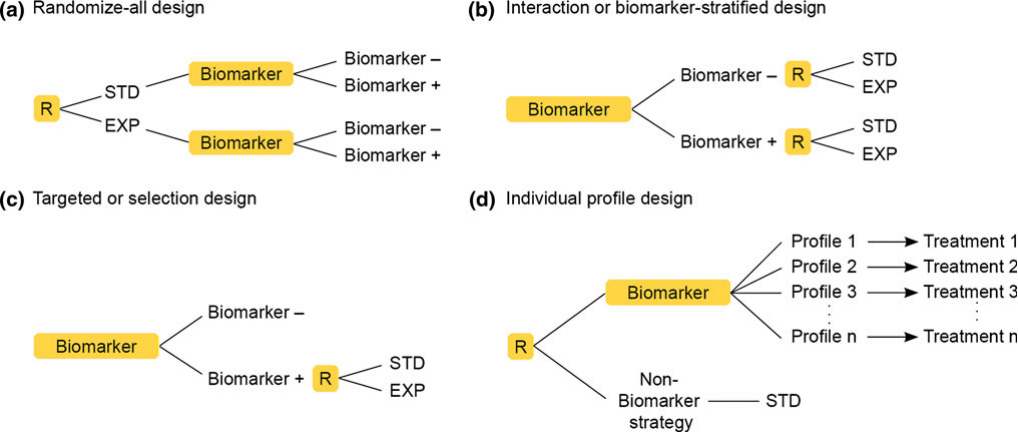
Common study designs for biomarker studies. **a** Traditional randomized-control design where the randomization (*R*) to standard (*STD*) or experimental (*EXP*) therapy is independent from the results of the biomarker test. A retrospective evaluation of this design is possible for DNA biomarkers. **b** So-called “Gold standard” design. Randomization to STD or EXP is performed in the total patient population stratified by the result of the biomarker results. **c** Restricted design. To reduce effort, only biomarker-positive patients are randomized to STD or EXP. Claims about the utility of a biomarker cannot be made from this trial alone. **d** Patients are randomized according to treatment based on biomarker or non-biomarker-dependent strategy. This is the most flexible design that provides information about specific individualized treatment rules according to, e.g., a DNA profile

In selection designs, patients are first tested with the companion test. Only test-positive patients are then randomized to treatment or control (Fig. 1c). As Buyse et al. (2011) observe, although these selection designs provide clear information about treatment efficacy, they fall short in substantiating the usefulness of the companion test, as they do not demonstrate lack of benefit in marker-negative patients. As soon as a test incurs costs, its usefulness for application should be quantified, and for this a selection design is only of limited value.

In the interaction or biomarker-stratified design (Fig. 1b), patients are first tested for the biomarker, and then randomized to treatment or control with stratification by the companion biomarker’s test result. This study design might be considered the gold-standard design for providing a sound basis for decision-making about the efficacy and benefit/risk of the experimental drug and the ability of the companion diagnostic to identify the appropriate patient population to be treated.

The interaction design allows for several hierarchical statistical testing procedures. The significance of the interaction test for the treatment effect in the test-positive and test-negative stratum may serve as a yardstick for the usefulness of the companion biomarker. If the interaction effect is significant, treatment effects in test-positive and test-negative patients differ; this difference alone does, however, not in itself assure the utility of a companion diagnostic. A small but significant treatment effect in test negatives, even if less marked than the effect in the biomarker-positive population, might still be of clinical importance.

Similarly, if the treatment effect in the larger population is small and irrelevant, the subgroup of biomarker-positive patients might be too small to achieve sufficient power in the interaction test. Evaluation of efficacy and benefit/risk should always consider the size of the estimated treatment effect as well.

Adaptive study designs offer a promising means to stop recruitment for futility in test-negative patients as soon as sufficient evidence about lack of efficacy in this subgroup is available. Strategies based on conditional power may be used to formalize adaptive study designs to some extent (Lachin 2005; Proschan and Hunsberger 1995; Schäfer et al. 2006). However, in the end, the assessment of the treatment effect size and the extent of benefit/risk must be set into perspective.

In instances where no gold standard are available, the utility of a diagnostic test may be evaluated by comparing biomarker-guided treatment with the standard, non-biomarker-based treatment selection. A highly simplified version of this is depicted in Fig. 1c with only two profiles and two treatments in the biomarker-guided treatment group. Again, significant differences between the outcome in the biomarker-guided and the conventional treatment group may be difficult to achieve and may fall short in demonstrating the efficacy of the experimental biomarker.

The individual profile design, which includes a large number of different profiles leading to the selection of one out of a large number of different treatments, is easily validated if it is planned and understood as a strategic trial comparing conventional treatment selection to an individualized decision rule (Fig. 1d). Even more complex approaches can be imagined. For example, an individualized therapy might combine several monotherapies, each selected based on the presence or absence of a specific DNA variant. Such designs and treatment plans reflect the paradigm of individualized treatment and personalized medicine. However, they pose new challenges to the regulatory system, requiring that the precise particularities of the application of drugs as mono-therapy, or in combination are well understood and substantiated with clinical trials data. Obviously, there is a gap between the validation of the biomarker-based treatment selection rule and the efficacy of the individual treatments to be applied based on a complex decision rule. Consideration needs to be given to the question of how this gap can be filled.

For example, the global statistics may demonstrate superiority of the individualized treatment selection when compared to the current standard. At the same time, some of the individual treatment combinations may seem to be inferior to the current standard. Here, it is clear that an upfront discussion is required to formulate the circumstances under which such inconsistencies can be ignored and under which the overall validity of conclusions has to be questioned.

Similarly, none of the many investigated treatments or treatment combinations may provide sufficient clinical information enabling the assessment of the safety of the suggested combination therapy in the way this is done in the standard drug development process, and we acknowledge that this standard process is justified by historical fallacies. Unless clear evidence is available that all drugs under investigation are safe and can be combined freely, a substantial amount of clinical data needs to be provided for each of the recommended treatments and treatment combinations. At least a basic assessment of safety is required because safety or benefit/risk may be different for different subpopulations identified with the biomarker rule. Along the line of the FDA definition of a companion test such information is a pre-requisite to use a complex decision rule in patient management. Of course, sample-size requirements will substantially increase with the number of treatments and treatment combinations under investigation, and the IPASS trial can be considered as a model for this.

In some instances, biomarkers may be investigated that are closely related to the mechanism of action of the companion drug, and in these instances it may be possible to provide the required information about the usefulness of the companion test as early as phase II drug development. In most instances, however, the traditional design—randomizing patients to treatment and control irrespective of biomarker outcome—may still be the optimal approach, such that overall superiority of a drug should first be demonstrated, subgroups defined by the companion test excluded from the labeling in retrospect, following the precautionary principle that harm does not need to be proven (Fig. 1a).

The credibility of this concept depends on the degree of independent validation that can be found in the different studies during the development program and the completeness of the sampling in every study. Convenience sampling in subpopulations of poorly defined origin should be avoided (Wang et al. 2010).

## Discussion

The three pillars of a physician’s work are diagnosis, therapy, and prognosis. Biomarkers form the basis for all aspects of personalized medicine, from “What does the patient have?” (diagnosis) to “What can the patient do about it?” (therapy) to “How bad is it?” (prognosis). Although the characteristics and applications of DNA biomarkers, DNA tumor biomarkers and general biomarkers differ substantially, the underlying methodological principles to validate each for use in clinical routine are identical.

DNA biomarkers are distinct from general biomarkers in several ways, such as protein expression levels, gene expression levels or even epigenetic biomarkers. Further, since germline DNA does not change over time, some studies utilizing biobanks may be interpreted as prospective biomarker studies under very special conditions. However, the critical aspect prior to marketing approval of a biomarker is how “convincing evidence” may be achieved and when it has been achieved. Novel strategies must be developed so that a biomarker assessed in biobanks can be considered validated, in particular if biobanks are to be used multiple times.

An important aspect of biobank biomarker studies is the transparency of the approach, which directly relates to the quality of reporting. In general, to date, reporting of biomarker studies has been weak. In a systematic review of studies published between 2004 and 2006, Fontela et al. (2009) evaluated diagnostic studies that used commercially available test kits for tuberculosis, HIV, and malaria, and found that *all* of the 238 articles fulfilling the study inclusion criteria had design issues. In only 10 % of the studies the reference standard was adequately described. Nine of the 25 indicators of the standards for Reporting of Diagnostic Accuracy (STARD) (Bossuyt et al. 2004) were reported in fewer than 25 % of the studies, even though the studies reviewed were published immediately following publication of the STARD statement. As Plint et al. (2006) have effectively demonstrated for therapeutic studies, if reporting guidelines are adopted by journals, and in consequence by authors, the quality of reporting is measurably improved. In addition, it is substantially simpler to read articles if authors follow reporting statements. Therefore, authors of biomarker studies are strongly encouraged to report their work using appropriate reporting guidelines, such as STARD, strengthening the reporting of observational studies in epidemiology–molecular epidemiology (STROBE-ME; Gallo et al. 2011), reporting recommendations for tumor marker prognostic studies (REMARK; McShane et al. 2005), reporting recommendations for OMICS studies (QUADOMICS; Parker et al. 2010) or the preferred reporting items for systematic reviews and meta-analyses (PRISMA, formerly QUORUM; Moher et al. 2009); for a complete list, the reader may refer to the Equator Network at http://equator-network.org.

While validation of diagnostic and prognostic biomarkers does not need to be linked to a specific therapy but rather to standard of care, validation of predictive biomarkers must be closely linked to the specific therapy under consideration.

In order to gain marketing approval, pivotal drug trials, i.e., randomized controlled phase III clinical trials generally operate from the paradigm recruiting a broad spectrum of patients, not only to achieve higher enrollment numbers, but also to investigate the generalizability of findings. If, however, a drug has different levels of efficacy and safety in different ethnicities, as in for example gemcitabine, tamoxifen (Sai and Saito 2011) or warfarin (El Rouby et al. 2004), it might be possible to leverage the power to detect efficacy and demonstrate safety of a drug as a rationale to conduct trials in ethnically homogenous populations. Although the ICH E5 guideline (European Medicines Agency 1998a) clearly outlines a framework to evaluate the impact of ethnic factors, the use of an ethnically homogeneous population to evaluate the effect of a biomarker might be more promising if the aim is detection of different effects between biomarkers (see the study of Link et al. 2008). If recruitment is done on a global level in a phase III clinical trial, one has to expect substantial population stratification, i.e., heterogeneity in the patient population, and as sample sizes per ethnic group are eventually small, identification of biomarkers might be hindered.

Even if a biomarker or a set of biomarkers has been identified to be a good diagnostic, prognostic, or predictive marker, application to clinical routine is not certain. Although predictive biomarkers may be readily applied, as specific therapies rely on biomarker results, clinical applications for diagnostic or prognostic biomarkers are more complex. Here, use in clinical routine will depend on the ease of applicability, including the media for biomarker measurement, and whether clinicians can measure the biomarker in their own laboratory, a local laboratory, or a specialized laboratory at some distance from their practices. Also important is the algorithm for obtaining the decision or a recommendation (Kruppa et al. 2012). If clinicians are able to perform calculations by hand and the rules are easy to interpret, acceptance of the biomarker(s) is more likely than if some kind of “black box” is required. Therefore, classifications and probabilities estimated by a logistic regression model are more likely to be accepted by clinicians than results obtained by machine learning methods, such as artificial neural networks or support vector machines—although these generally may look quite impressive.

We conclude that a priori planning of research strategies is vital for the identification and validation of biomarkers.
